# High Prevalence of Non-thyroidal Illness Syndrome in Patients with Chronic Obstructive Pulmonary Disease: A Systematic Review and Meta-analysis

**DOI:** 10.5152/eurasianjmed.2026.251290

**Published:** 2026-04-15

**Authors:** Pilar Fandos Vázquez, Jesús Díez-Manglano

**Affiliations:** 1 Department of Medicine, Dermatology and Psychiatry, University of Zaragoza, Spain; 2 University Hospital Miguel Servet, Zaragoza, Spain; 3 Internal Medicine Department, University Hospital Royo Villanova, Zaragoza, Spain

**Keywords:** Chronic obstructive pulmonary disease, meta-analysis, non-thyroidal illness syndrome, systematic review

## Abstract

Our aim was to estimate the prevalence of non-thyroidal illness syndrome in individuals with chronic obstructive pulmonary disease (COPD). A systematic review and meta-analysis of studies indexed in Embase, ScienceDirect, PubMed, Virtual Health Library, Web of Science, and Cochrane Library from inception through December 31, 2023, was performed. The protocol was registered in PROSPERO (CRD42024492108). Pooled prevalence and 95% CIs were calculated using the DerSimonian–Laird random-effects model, and heterogeneity was assessed with the *I*² statistic. Meta-regression analyses examined the influence of age, sex, and smoking status. Subgroup and sensitivity analyses were performed, and associations with arterial blood gas parameters (PaO₂, PaCO₂, and SaO₂) were evaluated using the inverse-variance method within a random-effects framework. Eleven studies including 1005 chronic obstructive pulmonary disease patients met the inclusion criteria. The overall pooled prevalence of non-thyroidal illness syndrome was 44.2% (95% CI, 30.0-58.4%) with high heterogeneity (*I* ^2^, 96.1%). Non-thyroidal illness syndrome was more common during acute exacerbations (54.5%) than in stable disease (13.8%). Meta-regression analyses showed that age, sex, and smoking status did not significantly account for heterogeneity. Among arterial blood gas parameters, a modest but statistically significant reduction in SaO₂ was observed in patients with non-thyroidal illness syndrome compared with those without (−2.65%, 95% CI, −4.56 to −0.74). No significant differences were detected for PaO₂ or PaCO₂. As a conclusion, non-thyroidal illness syndrome is a common finding in patients with COPD, particularly during acute exacerbations, and is associated with reduced oxygen saturation. These findings highlight the importance of monitoring thyroid function and oxygenation in this population.

**Main Points**

- This meta-analysis included 11 studies: 9 from Türkiye, 1 from Iran, and 1 from Nepal, involving a total of 1005 patients.
- Non-thyroidal illness syndrome (NTIS) occurs in 44.2% of patients with chronic obstructive pulmonary disease (COPD).
- The prevalence of NTIS was 46.1% in patients with acute COPD exacerbation and 22.9% in patients with stable COPD.
- Patients with COPD and NTIS have lower oxygen saturation than those with normal thyroid function.

## Introduction

Chronic obstructive pulmonary disease (COPD) is defined by a persistent and progressive reduction in airflow, driven by an amplified and long-standing inflammatory response in the airways and lung tissue to harmful particles or gases, including tobacco smoke and various forms of environmental or household pollution.^1^ Today, COPD is understood as a systemic disorder that extends beyond the lungs, involving multiple extrapulmonary manifestations and comorbidities that worsen quality of life, accelerate clinical deterioration, and negatively influence prognosis.^2^ ^-^^4^ Patients commonly experience acute exacerbations that result in emergency care visits and hospital admissions, further impair functional status, and raise mortality risk.^5^

Individuals with severe acute conditions often show decreased plasma triiodothyronine (T3) levels while maintaining normal or only slightly reduced thyroid-stimulating hormone (TSH) concentrations.^6^ This hormonal pattern, known as non-thyroidal illness syndrome (NTIS), has an unclear underlying mechanism but has been linked to worse clinical outcomes.^7^^,^^8^

The frequency and clinical impact of NTIS among people with COPD remain insufficiently explored. Reported prevalence rates vary widely, with some studies describing figures below 10% and others indicating that more than two-thirds of patients may be affected.^9^^,^^10^

The authors proposed that NTIS could be a common finding in COPD patients, both in stable conditions and during acute exacerbations. The purpose of this study was to assess how prevalent NTIS is in this population and to evaluate its clinical relevance.

## Methods

### Design

This systematic review was undertaken to estimate the prevalence of NTIS among individuals with COPD. The study protocol was registered in PROSPERO (CRD42024492108), and all analyses followed the Meta-Analysis of Observational Studies in Epidemiology (MOOSE) guidelines.

### Data Sources and Search Strategy

A comprehensive literature search was conducted in PubMed, Embase, Web of Science, the Cochrane Library, ScienceDirect, and the Virtual Health Library from database inception through December 31, 2023. The search strategy combined thyroid-related terms (e.g., hyperthyroidism, thyrotoxicosis, hypothyroidism, thyroid dysfunction, euthyroid sick syndrome, thyroxine, thyroid-stimulating hormone) with COPD-related terms (chronic obstructive pulmonary disease, chronic bronchitis, emphysema). Additional searches were carried out in ResearchGate and Google Scholar, and reference lists of eligible articles were manually screened to identify further relevant studies.

### Study Selection

Studies, encompassing conference abstracts, were included if COPD was diagnosed using spirometry and if they reported the prevalence of NTIS. Exclusion criteria comprised COPD diagnosed without spirometry, mixed respiratory populations in which COPD could not be isolated, publications in predatory journals, review articles, and studies that did not provide sufficient data for extraction. Titles and abstracts were screened independently by 2 reviewers. Full texts were retrieved when eligibility was unclear or when abstracts lacked essential information. Inter-reviewer agreement was quantified using the kappa statistic, and disagreements were resolved by consensus.

### Quality Assessment

Risk of bias was evaluated independently by both authors using the National Heart, Lung, and Blood Institute’s quality assessment tool for observational cohort and cross-sectional studies. The instrument assesses 14 methodological domains, including study aims, population definition, participation rate, sample size justification, exposure and outcome measurement, study duration, blinding of outcome assessors, follow-up, and control of confounding. Each study was rated as good, fair, or poor, with disagreements resolved through discussion.

### Data Extraction

The following variables were extracted from each eligible study: first author, publication year, country, study design, sample size, mean age, sex distribution, COPD status (stable or exacerbation), percentage of smokers, prevalence of NTIS, and reported values for PaO₂, PaCO₂, and oxygen saturation (SaO₂).

### Data Synthesis and Statistical Analysis

Statistical analyses were conducted with OpenMeta[Analyst], Review Manager (RevMan) 5.4, and R. Pooled NTIS prevalence was calculated using the DerSimonian–Laird random-effects model for binary outcomes, and results are presented with 95% confidence intervals. Differences in PaO₂, PaCO₂, and SaO₂ between patients with and without NTIS were examined using the inverse-variance method within a random-effects framework. Between-study heterogeneity was assessed using tau² and I² statistics, with high heterogeneity defined as *P* < .10 and *I*² > 50%. Publication bias was evaluated using funnel plots and Egger’s regression test.

### Subgroup and Sensitivity Analyses

Subgroup analyses were performed according to clinical status—stable COPD versus acute exacerbation. Sensitivity analyses included restricting the dataset to high-quality studies and conducting a leave-one-out meta-analysis to assess the robustness of pooled estimates.

## Results

### Study Selection

**Figure 1.**
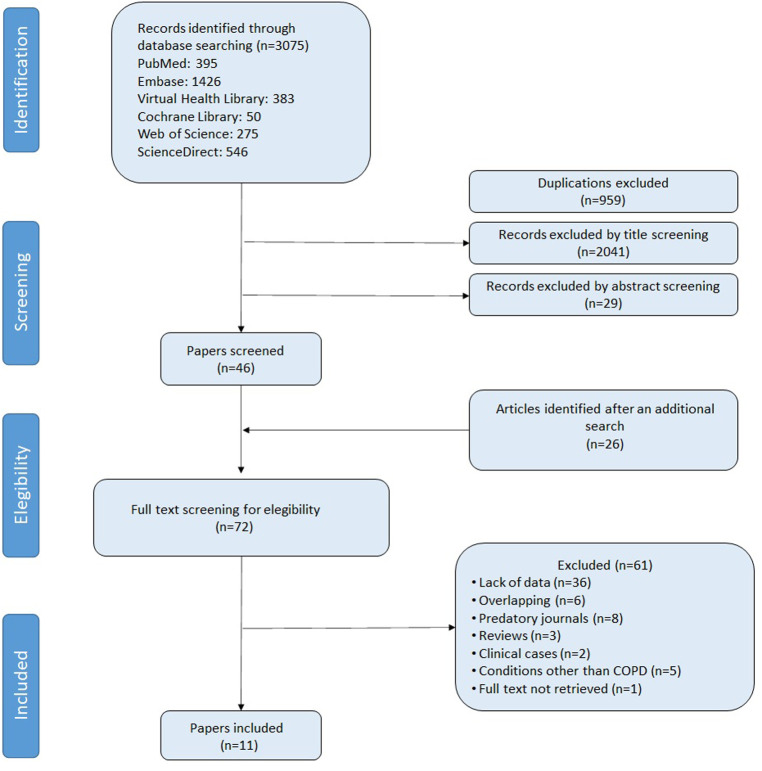
Flowchart of included studies.

The initial database search yielded 3075 records. Figure 1 illustrates the study selection process. Of these, 1426 records were retrieved from Embase, 546 from ScienceDirect, 395 from PubMed, 383 from the Virtual Health Library, 275 from Web of Science, and 50 from the Cochrane Library. After removing 959 duplicates, titles and abstracts were screened, resulting in the exclusion of 2070 records that were not relevant to the study objectives. This left 46 articles, to which 26 additional studies identified through manual searching were added. Following full-text review, 61 articles were further excluded (see Supplementary Material). Inter-rater agreement, as measured by the kappa statistic, was 0.726 for title screening and 0.798 for abstract screening. Ultimately, 11 cross-sectional studies met all inclusion criteria and were included in the analysis.^9^ ^-^^19^

### Study Characteristics

Table 1 summarizes the characteristics of the included studies, published between 2004 and 2023. Nine studies were conducted in Türkiye, one in Iran, and one in Nepal. Quality assessment classified 6 studies as good, 3 as fair, and 2 as poor, with full consensus among reviewers (Supplementary Table 1). Seven studies enrolled patients experiencing acute exacerbations, 2 included patients with stable COPD, and 2 involved both stable and exacerbated COPD populations. Only 6 studies reported data on %pFEV1, with mean values in all cases ranging between 30% and 50% (GOLD severe stage).

### Prevalence of NTIS in Chronic Obstructive Pulmonary Disease

**Figure 2.**
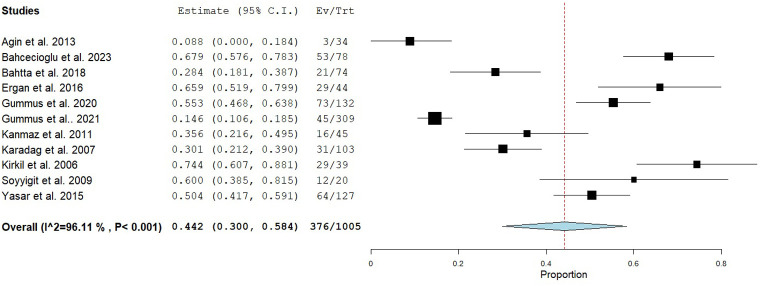
Forest plot of prevalence of non-thyroidal illness syndrome in COPD.

**Figure 3.**
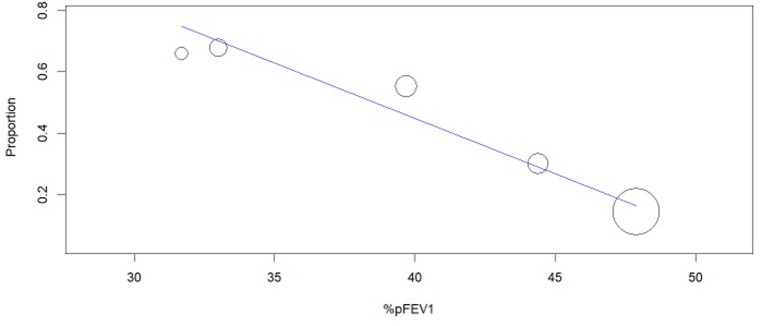
Meta-regression of non-thyroidal illness syndrome prevalence according to the mean %FEV_1_ in each study.

The 11 studies collectively included 1005 participants. The pooled prevalence of NTIS across all studies was 44.2% (95% CI, 30.0-58.4%; Figure 2), with substantial heterogeneity observed (*I*² = 96.1%). Meta-regression analysis indicated that mean age, proportion of female participants, study size (more or less than 100 participants), smoking prevalence, and NTIS definition did not account for the observed heterogeneity (Supplementary Table 2). However, mean %FEV_1_ could explain the heterogeneity (Figure 3), indicating that a lower %FEV_1_ was also associated with a higher prevalence of NTIS.

### Sensitivity Analysis

When restricting the analysis to studies rated as good quality, the pooled NTIS prevalence increased to 52.1% (95% CI, 39.1-65.1%). A leave-one-out analysis showed no significant changes in the overall estimates, confirming the robustness of the findings (Table 2).

### Non-Thyroidal Illness Syndrome Prevalence in Stable Vs. Acutely Exacerbated Chronic Obstructive Pulmonary Disease

**Figure 4.**
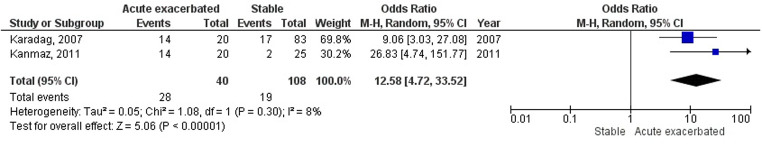
Forest-plot of non-thyroidal illness syndrome in patients with stable and acutely exacerbated chronic obstructive pulmonary disease.

NTIS prevalence was markedly lower in patients with stable COPD (13.8%, 95% CI, 9.1-18.4%) compared with those experiencing acute exacerbations (54.5%, 95% CI, 44.1-64.9%; Figure 2). Two studies, comprising 148 participants, directly compared NTIS prevalence between stable and exacerbated COPD, revealing a significantly higher prevalence among patients with acute exacerbations (odds ratio 12.58, 95% CI, 4.72-33.52; *P* < .001) with minimal heterogeneity (*I*² = 8%; Figure 4).

### Hypoxia and Hypercapnia

**Figure 5.**
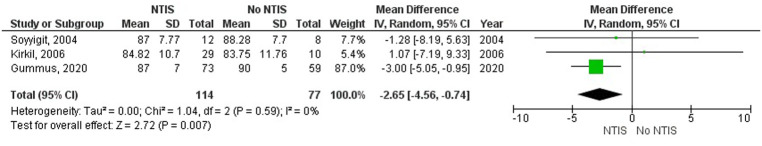
Forest plot of SaO_2_ in chronic obstructive pulmonary disease patients with and without non-thyroidal illness syndrome.

Five studies, all including patients with acute COPD exacerbations, reported data on PaO₂ and PaCO₂, and 3 reported SaO₂. No significant differences were observed between patients with and without NTIS in PaO₂ (−2.87 mmHg, 95% CI, −7.36 to 1.62) or PaCO₂ (−0.86 mmHg, 95% CI, −7.75 to 6.02; Supplementary Figure 1 and Supplementary Figure 2). However, a small but statistically significant reduction was seen in SaO₂ among patients with NTIS (−2.65%, 95% CI, −4.56 to −0.74; Figure 5).

Although Egger´s test did not indicate asymmetry, the limited number of studies and the funnel plots provided in the supplementary Figures 3, 4 and 5 suggested a potential publication bias.

## Discussion

Our study demonstrates 2 principal findings: nearly half of all individuals with COPD exhibit NTIS, and its occurrence during acute exacerbations is approximately twice as high as in the stable phase of the disease.

Two systematic reviews have previously explored thyroid function in COPD. In a meta-analysis of 9 studies involving 1307 participants, Agbor and colleagues reported that 45% of patients had some form of thyroid dysfunction, including 37% with hypothyroidism and 10% with hyperthyroidism.^20^ However, the reliability of these estimates is limited because 3 of the included studies came from predatory journals. Only 2 of the 9 studies provided data specific to NTIS. More recently, a larger meta-analysis including 25 studies and 3405 participants was published.^21^ This analysis showed that COPD is associated with lower TSH and T3 concentrations compared with the general population. Hormonal reductions were more pronounced during exacerbations. Consistent with these observations, earlier narrative reviews have suggested that NTIS is the most common thyroid abnormality seen in COPD.^22^^,^^23^

NTIS reflects a complex disturbance involving both central and peripheral resistance to thyroid hormones. Although its mechanisms remain incompletely characterized, alterations within the hypothalamic–pituitary–thyroid axis are believed to be central to its development. Illness-related changes in hypothalamic TRH, pituitary TSH, and peripheral hormone metabolism have been proposed.^24^ Inflammatory cytokines appear to play a central role by modifying deiodinase activity, leading to reduced type 1 and increased type 3 deiodinase function.^25^

Patients with COPD exhibit a chronic inflammatory state driven by oxidative stress.^26^ A vicious cycle of interrelation between inflammation, oxidative stress, and thyroid disorders, including NTIS, has been proposed.^27^ Additional COPD-specific factors may further predispose patients to NTIS. Multiple studies have demonstrated correlations between thyroid hormone levels and arterial blood gas values, with lower PaO₂ and higher PaCO₂ associated with reduced T3 concentrations.^10^^,^^17^ ^-^^19^ Hypoxia and oxidative stress may disrupt deiodinase activity and impair coenzyme Q10–dependent pathways.^28^^,^^29^ Galecka et al^30^ reported altered levels of deiodinase type 2 in COPD patients compared with controls. Coenzyme Q10, which participates in the conversion of thyroxine to T3, is often reduced in COPD, particularly during acute exacerbations.^31^ ^-^^33^ In the meta-analysis, PaO₂ and PaCO₂ did not differ meaningfully across groups, but a small—though potentially clinically relevant—difference in oxygen saturation (SaO₂) was observed. Because most included studies enrolled patients experiencing exacerbations, these findings should be interpreted cautiously.

Understanding the clinical significance of NTIS is essential. In several other conditions—such as ischemic heart disease, chronic kidney disease, and COVID-19—NTIS has been consistently linked to adverse outcomes, including increased mortality^34^ ^-^^37^ In a cohort of 125 COPD patients admitted to the intensive care unit, Yasar and colleagues found that NTIS was associated with longer intubation times and higher rates of pulmonary infection.^14^ However, the studies included in the meta-analysis did not report mortality or other robust clinical endpoints, limiting conclusions about prognosis in COPD specifically.

Whether NTIS should be treated remains an important clinical question. Current evidence does not support initiating thyroid hormone therapy for NTIS.^38^ In a study of COPD patients recovering from exacerbations, Kirkil et al^10^ observed spontaneous increases in free and total T3 levels as individuals transitioned back to clinical stability. To date, no randomized controlled trials have tested therapeutic interventions for NTIS in COPD, suggesting that the most evidence-based approach remains optimal management of the underlying pulmonary disease, both during exacerbations and in the stable state.

This review has notable strengths, including the inclusion of grey literature and the absence of language restrictions, which allowed for a more comprehensive examination of available evidence. Nonetheless, limitations must be acknowledged. Substantial heterogeneity was present across studies, likely influenced by small sample sizes and regional clustering. Most of the included research was conducted in Türkiye, where the prevalence of COPD and thyroid disorders varies considerably by region, raising concerns about generalizability.^39^ Additionally, diagnostic criteria for NTIS varied widely among studies—Gumus et al,^17^ for example, employed seven distinct classifications—which may have contributed to the wide range of prevalence estimates. This variability in the NTIS definition could limit the validity of the prevalence estimate.

In summary, the available evidence suggests that NTIS is common in patients with COPD, especially during acute exacerbations, although conclusions are constrained by methodological limitations, geographic concentration of the data, and potential publication bias. The relationship between NTIS, hypoxia, and hypercapnia remains uncertain. Further high-quality research is needed to clarify the clinical relevance of NTIS in this population. Until such data emerge, comprehensive and guideline-based management of COPD continues to represent the most appropriate clinical strategy.

## Supplementary Materials

Supplementary Material

## Figures and Tables

**Table 1.** Studies Included in Meta-Analysis

**Study**	**Year**	**Country**	**N**	**Mean Age, Years**	**Sex (Male/Female)**	**COPD Diagnosis**	**Mean %pFEV1**	**COPD Status**	**NTIS Definition**	**Smokers (%)**	**Quality**
Soyyigit et al^11^	2004	Türkiye	20	60	14/6	NR	NR	Exacerbated	T3 and/or T4 levels below lower limit and TSH levels within the normal limits	NR	Poor
Kirkil et al^10^	2006	Türkiye	39	64.8	34/5	GOLD	NR	Exacerbated	TT3 < 84 ng/dL and TSH 0.27-0.42 mUI/mL.	NR	Good
Karadag et al^12^	2007	Türkiye	103	66	103/0	GOLD	44.4	Stable (n = 83) and exacerbated (n = 20)	T3 and/or T4 levels below lower limit and TSH within normal limits or TSH within normal limits with T3 and/or T4 below lower limit	0	Good
Kanmaz et al^13^	2011	Türkiye	45	61.6	43/2	ATS	NR	Stable (n = 25) and exacerbated (n = 20)	T3 and/or T4 levels below lower limit and TSH levels within the normal limits	NR	Good
Agin et al^9^	2013	Iran	34	51.7	NR	GOLD	NR	Stable	NR	100	Fair
Yasar et al^14^	2015	Türkiye	125	NR	101/24	GOLD		Exacerbated	FT3 level below the lower limit, and/or FT4 level within the normal or low limits and TSH levels within the normal or low limits	NR	Good
Ergan et al^15^	2016	Türkiye	44	71.5	32/12	NR	31.7	Exacerbated	T3 levels below lower limit and TSH levels within or below the normal limits	20.5	Poor
Bahtta et al^16^	2018	Nepal	74	68	39/35	GOLD	NR	Exacerbated	NR	NR	Fair
Gumus et al^17^	2020	Türkiye	132	69.3	130/2	GOLD	39.7	Exacerbated	Seven categories: (1) Low T3, (2) Low T3 and low T4, (3) Low T3, low T4, and low TSH, (4) Low T4 (normal T3 and normal TSH levels), (5) High T4 (normal T3 and normal TSH levels), (6) High TSH (normal T3 and normal T4 levels), (7) Low TSH (normal T3 and normal T4 levels).	19	Good
Gumus et al^18^	2021	Türkiye	309	66	297/12	GOLD	47.9	Stable	NR	NR	Fair
Bahcecioglu et al^19^	2023	Türkiye	78	68.6	70/8	GOLD	33	Exacerbated	T3 levels below lower limit and TSH levels within or below the normal limits	NR	Good

ATS, American Thoracic Society; COPD, chronic obstructive pulmonary disease; GOLD, Global initiative of obstructive lung disease; NR, not reported; NTIS, non-thyroidal illness syndrome; pFEV1, predicted forced expiratory volume in one second; TSH, thyroid-stimulating hormone.

**Table 2.** Leave-One-Out Meta-Analysis

**Excluded Study**	**Estimated Effect** **Prevalence % (95%CI)**
Soyyigit et al^11^	42.8 (28.0-57.6)
Kirkil et al^10^	41.2 (27.1-55.3)
Karadag et al^12^	45.7 (29.8-61.6)
Kanmaz et al^13^	45.1 (29.8-60.3)
Agin et al^9^	47.8 (32.8-62.9)
Yasar et al^14^	43.6 (28.2-58.9)
Ergan et al^15^	42.1 (27.5-56.6)
Bahtta et al^16^	45.8 (30.2-61.5)
Gumus et al^17^	43.0 (28.1-58.0)
Gumus et al^18^	47.3 (34.0-60.5)
Bahcecioglu et al^19^	41.7 (27.6-55.8)

In all cases *P* < .001.
