# Supplementary material for: High Prevalence of Non-thyroidal Illness Syndrome in Patients with Chronic Obstructive Pulmonary Disease: A Systematic Review and Meta-analysis
Source: Eurasian J Med. 2026 Apr 15;58(2):e251290. doi: 10.5152/eurasianjmed.2026.251290 (PMC13185618; doi:10.5152/eurasianjmed.2026.251290)
Supplement: Supplementary Material [file supplementary_material.pdf]

Supplementary Material

Study or Subgroup	NTIS Mean	NTIS SD	NTIS Total	No NTIS Mean	No NTIS SD	No NTIS Total	Weight	Mean Difference IV, Random, 95% CI	Year
Soyyigit, 2004	57.35	12.5	12	56.95	16.7	8	9.0%	0.40 [-13.16, 13.96]	2004
Kirkil, 2006	57.93	16.2	29	50.65	13.62	10	13.8%	7.28 [-3.02, 17.58]	2006
Ergan, 2016	51.87	19.96	29	53.3	19.71	15	10.5%	-1.43 [-13.77, 10.91]	2016
Gummus, 2020	56	11	73	60	10	59	38.3%	-4.00 [-7.59, -0.41]	2020
Bahcecioglu, 2023	53.35	10.55	53	61.24	12.36	25	28.3%	-7.89 [-13.51, -2.27]	2023
**Total (95% CI)**			**196**			**117**	**100.0%**	**-2.87 [-7.36, 1.62]**	

Heterogeneity: Tau² = 10.33; Chi² = 7.01, df = 4 (P = 0.14); I² = 43%
Test for overall effect: Z = 1.25 (P = 0.21)

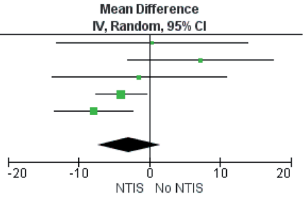

Supplementary Figure 1. Forest plot of PaO2 in chronic obstructive pulmonary disease patients with and without non-thyroidal illness syndrome

Study or Subgroup	NTIS Mean	NTIS SD	NTIS Total	No NTIS Mean	No NTIS SD	No NTIS Total	Weight	Mean Difference IV, Random, 95% CI	Year
Soyyigit, 2004	46.7	9.6	12	53.97	12.16	8	17.4%	-7.27 [-17.30, 2.76]	2004
Kirkil, 2006	50.78	19.03	29	40.23	8.92	10	18.9%	10.55 [1.69, 19.41]	2006
Ergan, 2016	72.33	17.62	29	75.03	19.71	15	15.2%	-2.70 [-14.56, 9.16]	2016
Gummus, 2020	45	12	73	42	9	59	25.6%	3.00 [-0.58, 6.58]	2020
Bahcecioglu, 2023	39.35	8.08	53	47.88	13.96	25	22.9%	-8.53 [-14.42, -2.64]	2023
**Total (95% CI)**			**196**			**117**	**100.0%**	**-0.86 [-7.75, 6.02]**	

Heterogeneity: Tau² = 44.75; Chi² = 18.39, df = 4 (P = 0.001); I² = 78%
Test for overall effect: Z = 0.25 (P = 0.81)

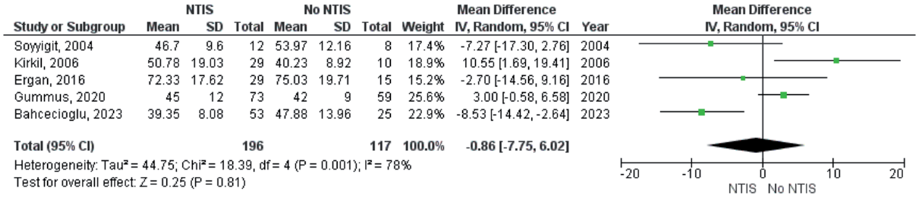

Supplementary Figure 2. Forest plot of PaCO2 in chronic obstructive pulmonary disease patients with and without non-thyroidal illness syndrome

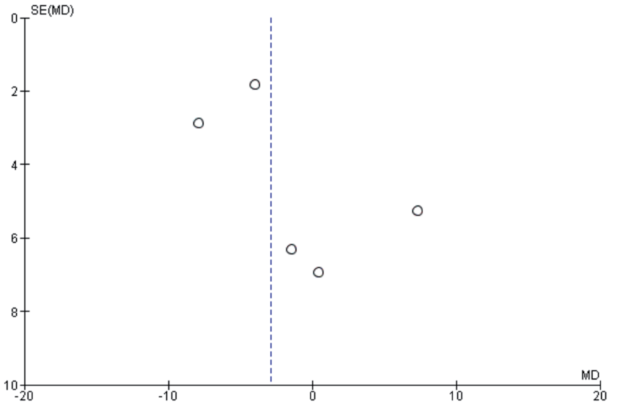

Supplementary Figure 3. Funnel plot of PaO2 in chronic obstructive pulmonary disease patients with and without non-thyroidal illness syndrome

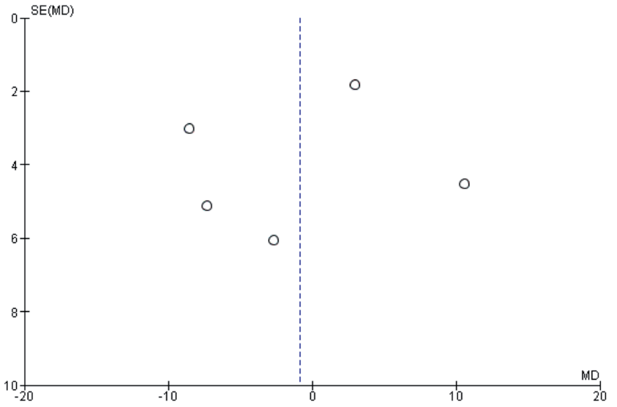

Supplementary Figure 4. Funnel plot of PaCO2 in chronic obstructive pulmonary disease patients with and without non-thyroidal illness syndrome

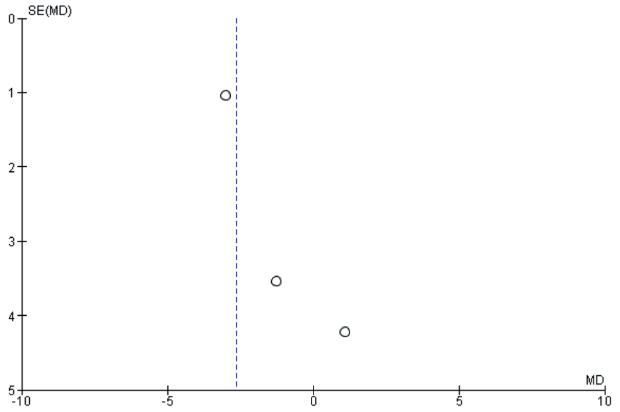

Supplementary Figure 5. Funnel plot of SaO2 in chronic obstructive pulmonary disease patients with and without non-thyroidal illness syndrome

Supplementary Table 1. Quality of the Included Studies

Study	Clearly stated objective	Defined population	Participation	Inclusion and exclusion criteria	Sample size	Exposure	Association	Level of exposure	Defined exposure measures	Exposure measured more than once	Clearly defined outcome measures	Blinded outcome assessors	Loss of follow < 20%	Confounding variables	Quality
Agin	Y	Y	NR	CD	N	Y	Y	N	Y	NA	N	NR	Y	N	Fair
Bahcecioglu	Y	Y	Y	Y	N	Y	Y	N	Y	NA	Y	NR	Y	Y	Good
Battha	Y	Y	Y	Y	N	Y	Y	N	Y	NA	N	NR	Y	N	Fair
Ergan	Y	Y	Y	Y	N	Y	Y	N	N	NA	Y	NR	Y	N	Poor
Gumus 2020	Y	Y	NR	Y	N	Y	Y	N	Y	NA	Y	NR	Y	Y	Good
Gumus, 2021	Y	Y	NR	Y	N	Y	Y	Y	Y	NA	N	NR	Y	Y	Fair
Kanmaz	Y	Y	Y	Y	N	Y	Y	N	Y	NA	Y	NR	NA	N	Good
Karadag	Y	Y	NR	Y	N	Y	Y	N	Y	NA	Y	NR	NA	N	Good
Kirkil	Y	Y	Y	Y	N	Y	Y	N	Y	NA	Y	NR	NA	N	Good
Soyyigit	Y	N	NR	Y	N	Y	Y	N	N	NA	Y	NR	Y	N	Poor
Yasar	Y	Y	NR	Y	N	N	Y	N	Y	NA	Y	NR	Y	N	Good

CD, cannot determinate; N, not; NA, not applicable; NR, not reported; Y, yes.

Supplementary Table 2. Meta-Regression Analysis of Factors that May Influence the Prevalence of NTIS in Patients with COPD

P

Covariables	Studies	Coefficient	95%CI	Mean age
10	0.021	−0.001, 0.042	.063	% female
10	0.001	−0.007, 0.010	.753	% smokers
4	−0.004	−0.008, 0.000	.067	%mean FEV_1
5	−0.036	−0.042, −0.030	<.001	Study quality
11	0.024	−0.145, 0192	.784	Study size (more or less 100)
11	−0.108	−0.363, 0.147	.408	NTIS definition
11	0.157	−0.081, 0.394	.196	

COPD, chronic obstructive pulmonary disease; NTIS, non-thyroidal illness syndrome.

References of the Excluded Studies

Studies Reporting Insufficient Data for Inclusion in the Meta-Analysis

S1. Gow SM, Seth J, Beckett GJ, Douglas G. Thyroid function and endocrine abnormalities in elderly patients with severe chronic obstructive lung disease. Thorax. 1987;42:520-5.

S2. Bratel T, Wennlund A, Carlström K. Impact of hypoxaemia on neuroendocrine function and catecholamine secretion in chronic obstructive pulmonary disease (COPD). Effects of long-term oxygen treatment. Respir Med. 2000;94:1221-8.

S3. Dimopoulou I, Ilias I, Mastorakos G, Mantzos E, Roussos C, Koutras DA. Effects of severity of chronic obstructive pulmonary disease on thyroid function. Metabolism. 2001;50:1397-401.

S4. Okutan O, Kartaloglu Z, Onde ME, Bozkanat E, Kunter E. Pulmonary function tests and thyroid hormone concentrations in patients with chronic obstructive pulmonary disease. Med Princ Pract. 2004;13:126-8.

S5. Mancini A, Corbo GM, Gaballo A, Valente S, Gigliotti P, Cimino V, et al. Relationships between plasma CoQ10 levels and thyroid hormones in chronic obstructive pulmonary disease. Biofactors. 2005;25:201-4.

S6. Sun B-J, Zou L, Liu C-T, Wang D-L. Comparison of thyroid hormone level in patients with chronic obstructive pulmonary disease at different stationary stages. Chinese J Clin Rehabil. 2005;9:100-1.

S7. Kaya E, Coşkun E. Evaluation of thyroid function tests in chronic obstructive pulmonary disease. Toraks Derg. 2007;8:211-4.

S8. Uzun K, Atalay H, Inal A. Thyroid hormon levels in patients with acute exacerbation of chronic obstructive pulmonary disease. Eur J Gen Med. 2007;4:80-2.

S9. Coşkun F, Ege E, Uzaslan E, Ediger D, Karadağ M, Gözü O. Evaluation of thyroid hormone levels and somatomedin-C (IGF-1) in patients with chronic obstructive pulmonary disease (COPD) and relation with the severity of the disease. Tuberk Toraks. 2009;57:369-75.

S10. Akbaş T, Karakurt S, Unlügüzel G, Celikel T, Akalin S. The endocrinologic changes in critically ill chronic obstructive pulmonary disease patients. COPD. 2010;7:240-7.

S11. Ciobanu L, Maciuc V, Costan R, Gorga A, Bobirca PA, Corduneanu S. Is COPD a risk factor for thyroid disorders? Eur Respir J. 2011;38:p3593.

S12. Mancini A, Corbo GM, Gaballo A, Raimondo S, Di Segni C, Gigliotti P, et al. Relationship between plasma antioxidants and thyroid hormones in chronic obstructive pulmonary disease. Exp Clin Endocrinol diabetes. 2012;120:623-8.

S13. Sobhy T, Mohamed Eiada T, Abou Zeid A, Mohamed Sharaf Eldin M. Hormonal dysfunction in patients with chronic obstructive pulmonary disease. Egypt J Chest Dis Tuberc. 2012;61:75-9.

S14. Abdel Dayem AM, Youssef SS, Mostafa MS. Assessment of thyroid gland functions and hypogonadism among male patients with COPD. Egypt J Chest Dis Tuberc. 2013;62:97-103.

S15. El-Yazed HA, El-Bassiony MRA, Eldaboosy SAM, El Gendi AEAA, Hashim M. Assessment of thyroid functions in patients with chronic obstructive pulmonary disease. Egypt J Chest Dis Tuberc. 2013;62:387-91.

S16. Madhuri G, Vardey SK, Nalin J, Dixit R. Evaluation of thyroid dysfunction in chronic obstructive pulmonary disease. Biomed Res. 2013;24:110-3.

S17. García-Olmos L, Alberquilla A, Ayala V, García-Sagredo P, Morales L, Carmona M, et al. Comorbidity in patients with chronic obstructive pulmonary disease in family practice: a cross-sectional study. BMC Fam Pract. 2013;14:11.

S18. Sarinc Ulasli S, Bozbas SS, Ozen ZE, Ozyurek BA, Ulubay G. Effect of thyroid function on COPD exacerbation frequency: a preliminary study. Multidisc Respir Med. 2013;8:64.

S19. Shoukry A, Said NS, Abd-Elrahman MNA, Saber T, Fawzy MA, Shalaby S. Thyroid dysfunction and inflammatory biomarkers in chronic obstructive pulmonary disease: Relation to severity and exacerbation. Egypt J Chest Dis Tuberc. 2013;62:567-74

S20. Aras G, Kanmaz D, Purisa S, Kadakal F, Yenturk E, Atabey F, et al. Are thyroid functions changing in patients with exacerbated COPD? J Acad Res Med. 2014;4:18-24.

S21. Prakash R, Suryanarayana V, Priya MP, Dharmic S. Evaluation of thyroid functions in chronic obstructive pulmonary disease. World J Med Sci. 2014;11:57-8.

S22. Weheed MA, Sultan KM, Obaidy WA. Spirometric tests and thyroid hormone concentrations in sample of Iraqi patients with chronic obstructive pulmonary disease. J Fac Med Baghdad. 2014;56:278-82.

S23. Vernekar P V, Ananda Vaidya K. Thyroid stimulating hormone and hemoglobin status in patients suffering from chronic obstructive pulmonary disease. Int J Sci Study. 2015;3:2013-6.

S24. Cheng Y, Dai L, Xia GG. The alterations and clinical significance of serum thyroid hormone levels in patients with acute exacerbation of chronic obstructive pulmonary disease. Chinese J Tuberc Respir Dis. 2016;39:939-43.

S25. Diao W, Shen N, Du Y, Sun X, Liu B, Xu M, et al. Identification of thyroxine-binding globulin as a candidate plasma marker of chronic obstructive pulmonary disease. Int J Chron Obstruct Pulmon Dis. 2017;12:1549-64.

S26. Chaudhary SC, Ahmad T, Usman K, Sawlani KK, Gupta KK, Verma AK, et al. Prevalence of thyroid dysfunction in chronic obstructive pulmonary disease patients in a tertiary care center in North India. J Fam Med Prim Care. 2018;7:584-8.

S27. De Blasio F, Dassetto D, Aredano I, Coni F, Ambrosanio R, Rella AM, et al. Sex differences in COPD comorbidities. Eur Respir J. 2018;52(Suppl 623):PA4040

S28. Xu CY, Guo, SG, Shen Y. Analysis of thyroid hormone levels in patients with chronic obstructive pulmonary disease during acute exacerbation. Fudan Univ J Med Sci. 2018;45:526-9.

S29. Galecka E, Kumor-Kisielewska A, Górski P. Association of serum deiodinase type 2 level with chronic obstructive pulmonary disease in the Polish population. Acta Biochim Pol. 2019;66:177-82.

S30. Ping H, Yang C, Li TS, Dai L, Xia GG, Zhang YJ. Changes in thyroid function in patients with acute exacerbation of chronic obstructive pulmonary disease and its clinical significance. Acta Medica Mediterr. 2019;35:1803-8.

S31. Yuksel A, Akpinar EE, Gulensoy ES, Ogan N, Alkan A. Assessment of thyroid function tests in COPD phenotypes. ERJ 2020;56(Suppl 64):543.

S32. Buklioska Ilievska D, Mickovski I. Comorbidities of patients with chronic obstructive pulmonary disease (COPD): thyroid abnormalities in stable COPD. Med Res J. 2021;6:204-10.

S33. Huang D, Wu D, He J, Chen M, Zhao X, Li D, et al. Association between thyroid function and acute exacerbation of chronic obstructive pulmonary disease. Int J Chron Obstruct Pulmon Dis. 2021;16:333-9.

S34. Alshamari AHI, Deli F, Kadhum HI, Kadhim IJ. Assessment of thyroid function tests in patients with chronic obstructive pulmonary disease. J Med Life. 2022;15:1532-5.

S35. Jafarinezhad A, Foumani AA, Tangestaninezhad A, Vaghari B. The association between subclinical hypothyroidism and exacerbation in patients with chronic obstructive pulmonary disease. J Curr Biomed Rep. 2022;3:1-6.

S36. Kim HJ, Park SJ, Park HK, Byun DW, Suh K, Yoo MH. Association of free thyroxine with obstructive lung pattern in euthyroid middle-aged subjects: A population-based study. PLoS One. 2022;17:1-11.

Studies with Duplicate or Overlapping Data

S37. Aras G, Kadakal F, Purisa S, Kanmaz D, Aynaci A, Isik E. Are we aware of restless legs syndrome in COPD patients who are in an exacerbation period? Frequency and probable factors related to underlying mechanism. Int J Chron Obstruct Pulmon Dis. 2011;8:437-43.

S38. Ergan Arava B, Ergün R. Sick euthyroid syndrome in severe COPD exacerbations. Eur Respir J. 2013;42(Suppl 57):P4755.

S39. Coskun F, Ege E, Uzaslan E, Ediger D, Karadag M, Gozu O. The relationship between thyroid hormones, somatomedin-C (IGF-1), anabolic hormones and the severity of disease in chronic obstructive lung disease (COPD). Eur Respir J. 2006;28(Suppl 50):290.

S40. Vijayaragavan R. A prospective assessment study of thyroid dysfunction in moderate to severe COPD. These. The Tamilnadu Dr. MGR Medical University. Chennai, Tamilnadu, India.

S41. Le TT, Simoni-Wastila L. Coprevalence of comorbidities in Medicare older patients with chronic obstructive pulmonary disease. Pharmacoepidemiol Drug Saf. 2020;29(Spec Issue):54-5.

S42. Shrma G, Saxena A, Varsha. A prospective study of thyroid dysfunction and clinical profile in COPD patients in a tertiary care centre. Lung India. 2022;39(Suppl 1):S118.

Predatory Journals

S43. Khalil OA, Yousif MM, Sadek AMEM, Khalifa A, Samir GM, Fawzi MS. Evaluation of thyroid dysfunction in patients with chronic obstructive pulmonary disease in medical intensive care unit of Zagazig University Hospitals. Int J Adv Res. 2016;4:270-81.

S44. Singh L, Jain A, Agrawal A, Tandon R, Kumar H. A study of prevalence of thyroid disorders in chronic obstructive pulmonary disease patients at a tertiary care center in U.P. Int J Contemp Med Res. 2016;3:1239-42.

S45. Singh V, Singh G. A study to find out correlation of thyroid hormone among COPD patients: A hospital based study. Int Arch Biomed Clin Res. 2019;5:6-8.

S46. Verma S, Gautam S, Bhan U, Vohra DK, Gupa A, Mittal S, et al. To study the association of thyroid dysfunction in chronic obstructive pulmonary disease patients (a cross sectional prevalence study single centre only). J Evol Med Dent Sci. 2019;8:2230-3.

S47. Vijayaragavan R, Prabhu A. A prospective assessment study of thyroid dysfunction in moderate to severe COPD. IOSR J Dent Med Sci. 2019;18:33-6.

S48. Sebasan RV, Baliga KV. Prevalence of thyroid dysfunction in moderate to severe chronic obstructive pulmonary disease patients – A cross-sectional study. Int J Sci Study. 2021;9:160-3.

S49. Meena R, Khangarot S, Meena A, Sharma A, Ranjan A, Sharma G, et al. A prospective study of thyroid dysfunction and clinical profile in COPD patients in a tertiary care centre. Int J Curr Res. 2022;14:21199-203.

Review Articles

S50. Milkowska-Dymanowska U, Bialas AJ, Laskowska P, Górski P, Piotrowski WJ. Thyroid gland in chronic obstructive pulmonary disease. Adv Respir Med. 2017;85:28-34.

S51. Matsegora NA, Shpota OE. The pathogenetic basis of correlation secretion thyroids hormons with progress and prognosis chronic obstructive pulmonary disease (COPD). J Educ Health Sport. 2018;8:525-33.

S52. Akpinar EE. An underestimated comorbidity of COPD: Thyroid dysfunction. Tuberk Toraks. 2019;67:131-5.

S53. Sukholytka M, Hypothyroidism and chronic obstructive pulmonary disease. Miznar Endocrinol Z. 2020;16:643-7.

Clinical Cases

S54. Ezrin C, MacLachlan M, Walter EP, Volpe R. «"Iodide-myxedema"» in patients with chronic chest disease. Can Med Assoc J. 1961;85:287-9.

S55. Butland RJA, Pang JACK, Geddes DM. Thyroxine and dyspnoea in emphysema Br J Dis Chest. 1981;75:96-7.

Studies Involving Patients with Conditions Other than COPD, COPD-Associated Infections, or Concomitant Thyroid Disorders

S56. Bacakoglu F, Basoglu OK, Gürgün A, Bayraktar F, Kiran B, Ozhan MH. Can impairments of thyroid function test affect prognosis in patients with respiratory failure?. Tuberk Toraks Dergisi. 2007;55:329-35.

S57. Nafae RM, Mohammed MA, Morsi AF, Ibrahim DA. Thyroid function in respiratory failure patients. Egypt J Chest Dis Tuberc. 2014:63:513-21.

S58. Terzano C, Romani S, Paone G, Conti V, Oriolo F. COPD and thyroid dysfunctions. Lung. 2014;192:103-9.

S59. Sivaranjani H, Chaitra KR. The study of pulmonary function in patients with hypotiroidism. Int J Adv Med. 2019;6:1774-8.

S60. Zhang Y, Liu H. Changes of thyroid hormones in patients with COPD and infection and the correlation with low T_3 syndrome. Lab Med. 2022;37:1062-5.

Full Text Not Retrieved

S61. Pechatnikov LM. [Significance of hypophyseo-thyroid disorders in chronic obstructive bronchitis]. Klin Med (Mosk) 1989;67:40-3.
